# Robust visual detection of brake-lights in front for commercialized dashboard camera

**DOI:** 10.1371/journal.pone.0289700

**Published:** 2023-08-11

**Authors:** Jiyong Moon, Seongsik Park

**Affiliations:** Department of Artificial Intelligence, Dongguk University, Seoul, Korea; University of Lagos Faculty of Engineering, NIGERIA

## Abstract

The collision avoidance system (CAS) is an essential system for safe driving that alerts the driver or automatically applies the brakes in an expected situation of a vehicle collision. To realize this, an autonomous system that can quickly and precisely detect brake-lights of preceding vehicle is essential and this should works well in various environments for safety reason. Our proposed vision algorithm solves these objectives focusing on simple color features rather than a learning algorithm with a high computational cost, since our target system is a real-time embedded device, i.e., forward-facing dashboard camera. However, the existing feature-based algorithms are vulnerable to the ambient noise (noise problem), and cannot be flexibly applied to various environments (applicability problem). Therefore, our method is divided into two stages: *rear-lights region detection* using gamma correction for noise problem, and *brake-lights detection* using HSV color space for applicability problem, respectively. (i) *Rear-lights region detection*: we confirm the presence of the vehicle in front and derive the rear-lights region, and used non-linear mapping of gamma correction to make the detected region robust to noise. (ii) *Brake-lights detection*: from the detected rear-lights region, we extract color features using the HSV color range so that we can classify brake on and off in various conditions. Experimental results show that our algorithm overcomes the noise problem and applicability problem in various environments.

## Introduction

Whether it is used as a driver assistance system or as a component of autonomous driving, one of the most important factors for safe driving is the collision avoidance system (CAS) [1]. CAS recognizes a collision in advance and avoids it or mitigates the damage from it. Therefore, CAS must recognize the situation around the vehicle and infer information from it. When a collision is expected, the system should either send a warning to the driver or act autonomously without any driver input, i.e., by automatically applying the brakes [2]. In particular, CAS is important in rear-end collision, one of the most frequent traffic accidents [3]. The advanced CAS detects and responds to danger in a rear-end collision situation based on distance information using distance measuring sensor or surrounding context information using vision sensor.

Distance measuring sensors, *e.g*., radars and LiDAR, and their algorithms [4–6], demonstrates significantly high performance; however, it is too expensive to be commercialized [2]. But more importantly, the sensor-based method has a disadvantage in rear-end collision; they are operated by estimating the distance to the vehicle in front [7]. Therefore, the distance from the vehicle in front must be sufficiently close or close at a fast speed for the sensor-based CAS to recognize and respond to a rear-end collision situation in advance. This constraint makes it difficult for the sensor-based method to respond flexibly in the rear-end collision situations. Furthermore, there is a possibility of missing the right moment when it should have responded more quickly.

On the other hand, vision sensors installed in vehicles and their intelligent methods can see, detect, and distinguish objects directly, owing to state-of-the-art lane detection, vehicle detection, and object identification technologies in computer vision [8]. Especially, vision-based method is much more suitable for the rear-end collision situation than the sensor-based method, because it is independent to distance information; it can acquire high-level information, such as the lane to which the vehicle in front belongs and whether the brake of the vehicle in front is on or off through the optical camera. This high-level information can help predict the braking state of the vehicle in front more accurately and flexibly, allowing the vision-based method to cope with rear-end collision situations more efficiently than the sensor-based method.

In accordance with the above, we propose a vision-based algorithm to efficiently cope with rear-end collision situations that can maximize the strength of the vision-based approach. Various vision-based algorithms have been proposed. Some of these methods use machine learning or deep learning algorithms for the detection of rear-lights region and brake-lights [9–11]. These methods perform the detection process by extracting spatial information from the input image. Although these learning algorithms show relatively good performance, they are difficult to use in real-time CAS because of their large computational cost [2], especially in the case of deep learning [1]. Additionally, learning-based methods require considerable training time along with the acquisition/annotation of images/videos to train the model. These burdens may be greater depending on the model size [12]. Therefore, we propose an algorithm based on basic features such as color to operate in real-time and do not require model training (feature-based method).

Feature-based methods implement rear-lights region detection and brake-lights detection algorithms based on simple color features or brightness intensity [13–19]. They first extract red color features from a specific color space to find the rear-lights region based on the characteristic that rear-lights are being red. From the extracted color features, they conduct a verification process based on the symmetric characteristic of the rear-lights to find the optimal rear-lights pair [9, 20]. In the detected rear-lights region, the vehicle’s brake operation is finally determined based on color features or brightness intensity. However, although these feature-based methods are portable and efficient, they are highly vulnerable to surrounding noise [10, 18, 21, 22]. Input images may contain a significant noise depending on the intensity of light, surrounding objects, and weather conditions. In this case, it is challenging to extract features from the image. It may be effective at nighttime when rear-lights region and brake-lights of cars are clearly identified. However, ambient lights, such as streetlights, may act as noise even at nighttime, resulting in distorted results if proper preprocessing to eliminate noise fails. Optimal rear-lights region verification process such as symmetry test and the size-shape test may be distorted if the features are noisy because these methods are mainly conducted after selecting candidate objects with features extracted from the color space. In feature-based methods, controlling for surrounding noise is important because performance depends on the effectiveness of the extracted features, but existing feature-based methods do not explore noise processing in depth beyond simple noise filtering or morphological operations. In addition, previous feature-based methods mainly evaluated performance at nighttime or daytime. However, we argue that the method should be evaluated in more diverse environments to evaluate the practical applicability of the proposed rear-lights region detection and brake-lights detection algorithms. In summary, the existing feature-based algorithms are sensitive to the effects of ambient noise (noise problem), and cannot be flexibly applied to various environments (applicability problem).

**Fig 1:**
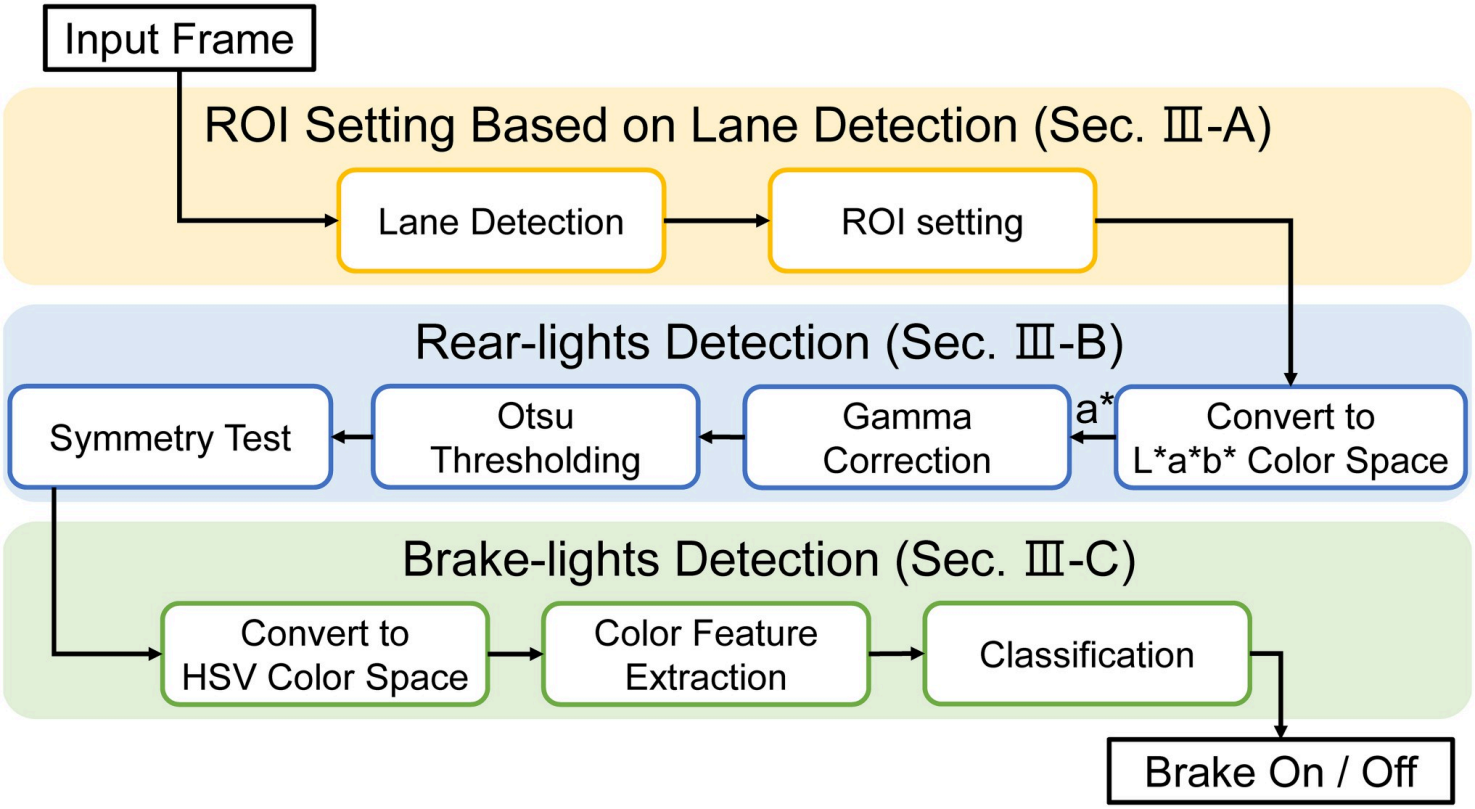
The proposed algorithm.

Therefore, we propose additional techniques to enhance the noise robustness and environmental applicability of the algorithm. The overall algorithm is shown in Fig 1. First, an ROI was established using a lane detection algorithm to consider only vehicles in front of the same lane. Setting an appropriate ROI not only reduces unnecessary noise in the input, but also can contribute to performance improvement by focusing attention on key parts. Second, rear-lights region detection is performed to check the presence of a vehicle in front and to find optimal rear-lights pair for brake-lights detection. In this process, we use gamma correction for noise reduction. Non-linear mapping of gamma correction guarantees consistent rear-lights region detection performance in various environments by reducing the effect of noise and making the key object stand out. Finally, based on the detected rear-lights region, the algorithm detects and interprets the preceding vehicle’s brake-lights to determine whether the brakes are operating to prevent rear-end collisions. In this process, classification is made based on the HSV color range. The HSV color range we found from the training samples provides a global decision threshold that can be applied in various environments.

Our main contributions can be summarized as follows:

(1) We design the algorithm based on simple features to avoid the high computational cost of the learning-based algorithm.
(2) We propose a noise reduction technique based on gamma correction to solve the noise problem faced by existing feature-based algorithms. Non-linear mapping of gamma correction reduces noise in color channels and makes the key object stand out.
(3) We propose HSV color range filtering to improve the applicability of feature-based brake-lights detection. The filtering range found from a small number of training samples can be applied in various environments including daytime and nighttime.
(4) Extensive experimental results show that our proposed algorithm effectively improves the noise problem and the applicability problem. Our evaluation scenarios include daytime and nighttime as well as cloudy and rainy conditions. Since each environment has different noise and lighting conditions, we demonstrate the high practical applicability of proposed rear-lights region detection and brake-lights detection methods as well as the noise reduction technique.

## Related work

### Rear-lights region detection

In most cases, rear-lights detection is performed first for vehicle detection [9, 20–28] or brake-lights detection [10, 11, 13–19, 29]. Among them, in feature-based methods, rear-lights regions are mainly detected by extracting color features from a specific color space (*i.e*., RGB [9, 14, 15, 17, 20, 27], YCrCb [10], L*a*b* [22], HSV [21, 23, 28], and YUV [19]) based on their characteristics of being red. Therefore, some studies focus on detecting rear-lights at nighttime, when red color features can stand out against a dark background [14, 17, 20, 21, 23, 27, 28]. After color space conversion of the given image, rear-lights candidate regions are extracted by applying thresholding to the channel containing red color components. Morphological operations [17, 23, 28] or noise filtering [14, 21] may be conducted to remove surrounding noise. Then, verification methods such as symmetry test [20, 21, 23] and aspect ratio test [17, 21] are used to determine the optimal rear-lights region based on the symmetrical characteristics of the rear-lights. This process is almost the same when detecting the rear-lights region at daytime when there is more surrounding noise [9, 10, 15, 19, 22]. Recently, deep learning or machine learning based methods have been proposed for rear-lights region detection. Some of these learning-based methods introduce trained models as part of rear-lights region detection [13, 16, 18, 24, 29, 30]. They use a learning model in performing vehicle detection to improve the accuracy of rear-lights region detection. Adaboost classifier [13, 18], cascade classifier [24], or deformable part model (DPM) [29] is trained to detect the rear of vehicles from given images. The rear-lights region is searched in a space confined to the rear of the vehicle, making detection easier. Conversely, Adaboost classifiers are also used to determine optimal pairs for rear-lights candidate regions found in a feature-based manner [16, 30]. In some methods, the entire process of rear-lights region detection is conducted by the learning model [11, 25]. They either use different models (*i.e*., R-CNN [31] and FCN [32]) to perform vehicle detection and rear-lights region detection [11], or use a single model (*i.e*., CNN-LSTM [33, 34]) to implement a one-stage rear-lights region detection process [25].

### Brake-lights detection

Brake-lights detection refers to methods that determine whether the brakes of vehicles operate, which is more challenging than simply detecting the rear-lights region. In feature-based methods, brake-lights detection is conducted following rear-lights region detection [13–19]. Color features are also useful for brake-lights detection [14, 18, 35, 36]. They determine whether the vehicle’s brakes operate based on the red color intensity of the rear-lights region in a defined color space. Besides color features, brightness intensity can also be used for brake-lights detection [13, 15, 19, 35]. Since the brightness of the rear-lights region is generally greater in brake-on situations than in brake-off situations, they compare the brightness intensity of the rear-lights region to a certain threshold to determine whether the vehicle’s brakes operate. In addition, brake-lights detection is also conducted based on the ROI size of the rear-lights region [17] or the frequency domain [16]. A number of learning-based methods have been proposed for brake-lights detection [10, 11, 29, 37–42]. Some methods extract color features from the derived rear-lights region or vehicle region, and train a classifier that predicts braking conditions with the extracted features [10, 11, 29, 38]. By fine-tuning pre-trained object detectors (*i.e*., YOLO [43, 44], Mask R-CNN [45]), they recognize vehicle braking conditions directly from a given image [37, 39–42].

### Discussion

As mentioned earlier, we propose a feature-based rear-lights region detection and brake-lights detection algorithms. Our rear-lights region detection algorithm is similar to previous feature-based methods [9, 10, 13, 14, 17, 19–23] in that it finds candidate rear-lights regions through color channel thresholding. However, they rarely attend to noise reduction and only apply simple filtering or morphological operations to color channels. On the other hand, we apply a noise reduction technique based on gamma correction before conducting thresholding to reduce noise in color channels and improve the accuracy of rear-lights region detection. We use the symmetry test [20, 21, 23] and aspect ratio test [17, 21] to find the optimal rear-lights pair, but it should be noted that we conduct verification on rear-lights candidate regions where noisy blobs are effectively removed. In addition, our brake-lights detection method is similar to previous methods [14, 18, 35, 36] in that it uses color features, but we use HSV color range filtering to improve the applicability of the algorithm. Color features extracted by HSV color range filtering can be used as global decision values under various environmental conditions, unlike previous methods limited to nighttime [14, 18] or daytime [13, 35].

## Proposed method

This section presents an algorithm that performs the detection of rear-lights region and brake-lights in a given image. The algorithm was divided into three steps. First, ROI based on lane detection was set to match the rear-end collision situation. Second, noise-robust detection of rear-lights region was performed using gamma correction and L*a*b* color space. Finally, detection of brake-lights was performed based on the color features extracted from the HSV color space.

In accordance with bioethics and safety act in the ministry of health and welfare (MOHW), Korea, this study does not collect or record personally identifiable information, does not experiment with human subjects, and uses only observation equipment that does not cause physical changes, hence, this study is not subject to IRB review.

### ROI setting based on lane detection

Only information from the vehicle in front should be considered to deal with rear-end collision situations. Therefore, the algorithm should focus only on the information of the car in front by setting an appropriate ROI. Furthermore, setting the ROI can make the algorithm much more efficient by reducing noise and excluding unnecessary processing [13]. Some approaches set the ROI arbitrarily in this process. However, the position of the preceding vehicle may vary from frame to frame depending on road conditions, and in this case, applying the same ROI to each frame is suboptimal. Therefore, we generate the ROI based on the lane detection algorithm to set the ROI adaptive to the road condition of the input frame. The lane detection algorithm we used can simply and efficiently derive lanes on the actual road through the forward monocular camera of the vehicle using techniques such as edge detection, orientation filtering, and connected component analysis [46]. Since lane detection is out of our main research scope, please see the reference for more details on the lane detection algorithm we used. In addition to the algorithm we referenced, other lane detection algorithms can also be used for the same purpose.

**Fig 2:**
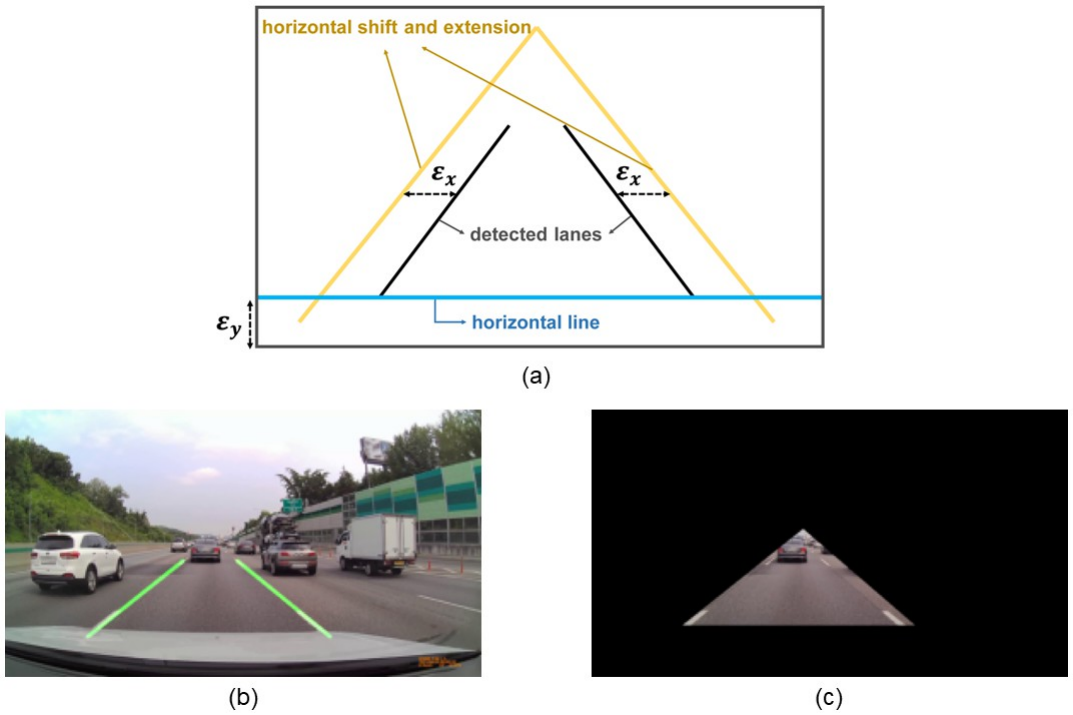
ROI setting result based on lane detection algorithm. (a) Transformation method. (b) Result of applying the lane detection algorithm. (c) Result of masking operation based on the derived ROI.

Using the lane detection results, we set up an adaptive ROI to consider only the cars ahead in the same lane. Fig 2 shows the entire process. As shown in Fig 2(b), the lane detection algorithm was applied to the input image and two suitable lanes were found as a result of it. Transformation was applied to the derived two lanes in the manner shown in Fig 2(a). The left and right lanes are shifted by *ϵ*_*x*_ and extended to form two refined lines. In addition, a horizontal line is added at a position *ϵ*_*y*_ apart from the bottom of the image. Finally, the three lines form a triangle, which becomes our ROI mask for extracting only the information of the current lane. We set *ϵ*_*x*_ = 30, *ϵ*_*y*_ = 0.4 * *w*_*h*_, where *w*_*h*_ is the height of the input image. The final result to which the generated triangular ROI mask was applied is shown in Fig 2(c). This process improved the efficiency of the algorithm by filtering out redundant information and allowing only information about the preceding vehicle to be considered. In addition, since we use lane detection to generate ROIs that are adaptive to road conditions, our ROI settings are flexible for input frames and various environments.

### Rear-lights region detection

Detecting the rear-lights region is essential for brake-lights detection in the feature-based method. Therefore, we localize the rear-lights region based on a simple color feature, but introduce a novel method to effectively remove the surrounding noise.

#### Color space

Red-color features were mainly used in several studies for the detection process of rear-lights region, focusing on the red color. Therefore, various color spaces such as RGB [9, 14, 17, 29], YCrCb [10], L*a*b* [13, 22], HSV [21], and YUV [19] were used. Among them, L*a*b* color space can approximately match the color difference that the human eye can detect [22]. Additionally, color features can be extracted less sensitively to changes in light by separating the L* channel containing the luminance information and the a* channel and the b* channel representing the color component [13]. Therefore, we derived the rear-lights region of the vehicle using the a* channel representing the red and green components in the L*a*b* color space, besides using the red-color property of the rear-lights.

#### Gamma correction and threshold method

**Fig 3:**
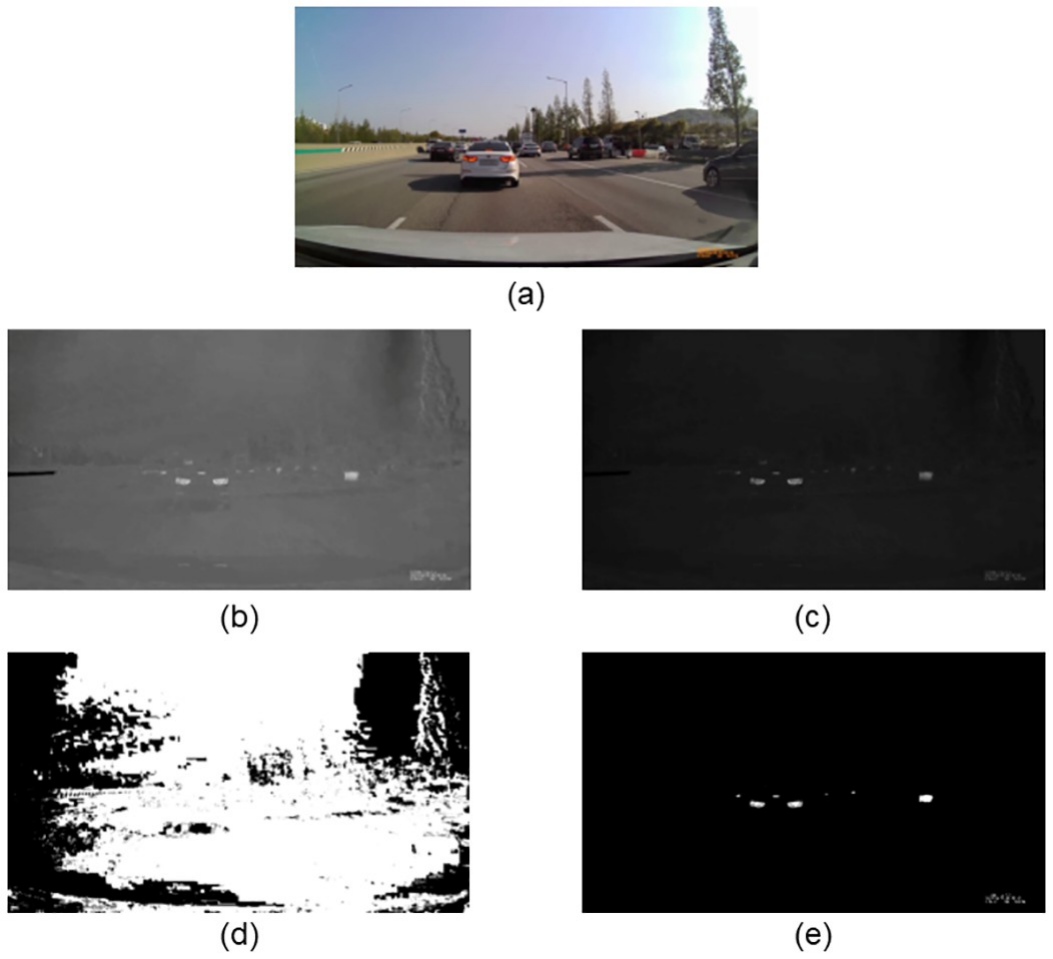
Result of color space conversion and thresholding. (a) Original image. (b) a* channel after converting the original image into L*a*b* color space. (c) Result of applying the gamma correction with *γ* = 10 to the original a* channel. (d) Result of applying threshold method directly to the a* channel of the image. (e) Result of applying threshold method to a* channel to which gamma correction is applied.

**Fig 4:**
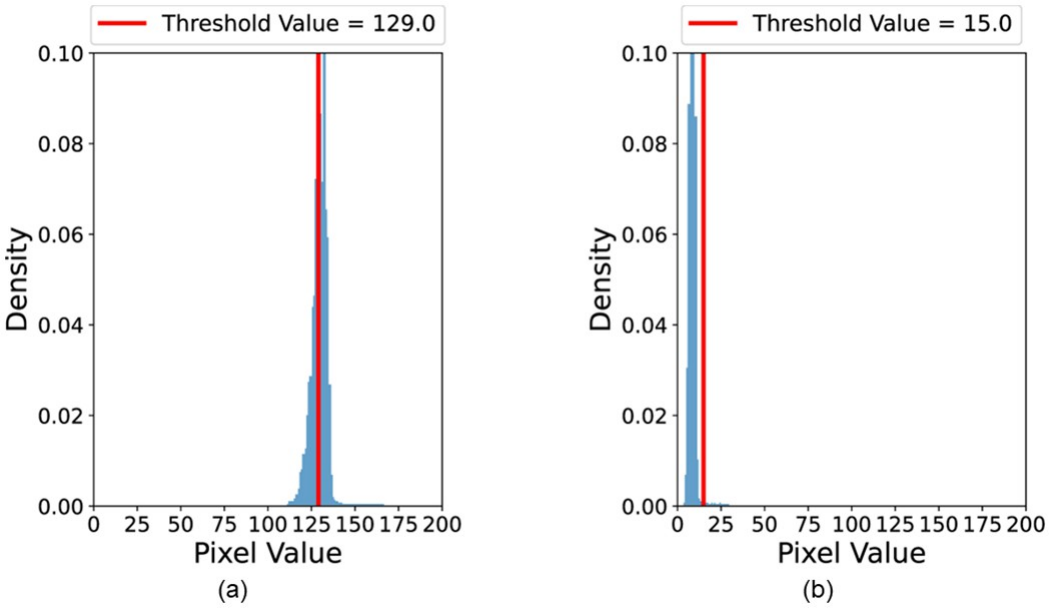
a* channel histogram and threshold value when the Otsu threshold method is applied. (a) When no preprocessing is applied. (b) When gamma correction is applied.

The region in the a* channel, after converting the given image in Fig 3(a) into the L*a*b* color space, has a high pixel intensity owing to the strong red component of the rear-lights region of the vehicle (see Fig 3(b)). Therefore, we expect that the rear-lights region of the vehicle can be effectively derived if thresholding is applied to the corresponding a* channel. We use the Otsu threshold method, which finds the optimal threshold value that minimizes the intra-class variance of the two groups, that is, maximizes the inter-class variance of the two groups [47]. The biggest advantage of the Otsu threshold method is that it automatically finds the optimal threshold value that can distinguish the background from the object in a given image [10]. It is highly preferable to set an adaptive threshold value for each image rather than setting a global threshold value because it is necessary to extract the region of rear-lights in various environments, such as daytime or nighttime. The result of applying thresholding to the a* channel is shown in Fig 3(d). Contrary to existing expectations, the binary image created as a result of thresholding contained a lot of noise; moreover, it also failed to extract the rear-lights region. This is because the region of rear-lights in the a* channel has strong pixel intensity, but several regions of surrounding noise, including sunlight and background, also have sufficient pixel intensity. This can also be confirmed through Fig 4(a), which shows the histogram of the original a* channel and the threshold value after applying the Otsu threshold method. The left side area was considered as the background by the Otsu method, and the right side area was considered as the object to be extracted based on the threshold value indicated by the red line in the histogram. Most of the values are concentrated around pixel value of 125, and in this case, the Otsu threshold method does not conduct as we expected. Dense pixel distribution causes the inter-class variance to be small at any threshold value, blurring the boundary between the target object and noise blobs. In order for the Otsu threshold method to distinguish the two groups of pixels well, a clear difference between the two groups must be made in the pixel distribution (*i.e*., bimodal distribution). Therefore, additional preprocessing was required for the threshold algorithm to effectively extract the region of rear-lights.

We propose a noise reduction technique based on gamma correction so that the pixel intensities between the target rear-lights region and the surrounding noise region can be clearly distinguished. Gamma correction is one of the image contrast enhancement techniques. Gamma correction is used to improve the overall brightness and contrast of the image by converting the existing pixel values of the image through non-linear mapping using the parameter *γ* [48]. The mapping function is as follows:

$$\begin{array}{c} x^{\prime }=T\left(x\right)=255\times {\left(\frac{x}{255}\right)}^{\gamma } \end{array}$$ (1)

**Fig 5:**
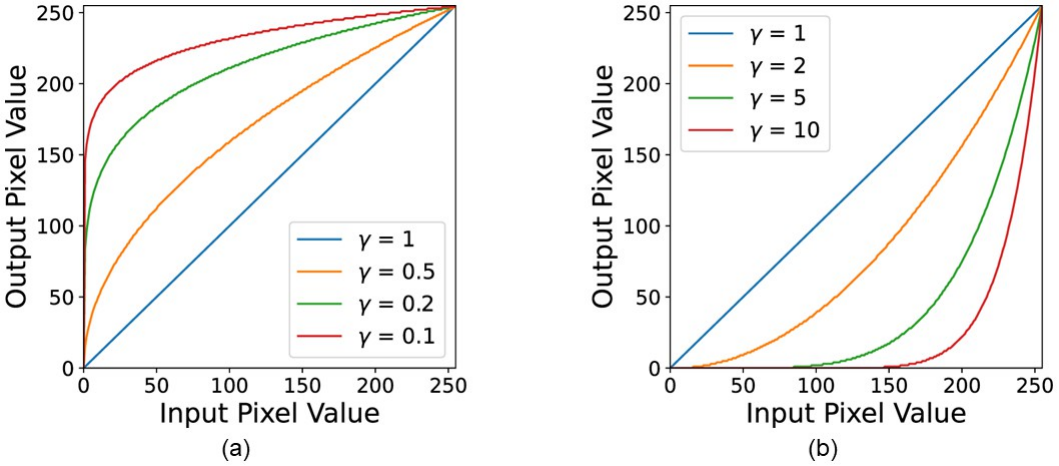
Gamma correction mapping curves. (a) Gamma correction mapping curve when 0 < *γ* ≤ 1. (b) Gamma correction mapping curve when 1 ≤ *γ*.

In Eq (1), *x* is the original pixel value, and *x*′ is the new pixel value converted by the gamma correction mapping function *T*. The change of pixel value according to *γ* can be seen in Fig 5. In general, gamma correction increases image brightness and contrast when 0 < *γ* < 1, as shown in Fig 5(a). Additionally, the pixel value mapping has a non-linear structure that occurs larger as the pixel value is lower, and as the *γ* approaches 0, the change occurs more rapidly in a wider range of pixel values. However, the image became dark when 1 < *γ* (see Fig 5(b)) Similarly, a sharp decrease in pixel value was seen in a wider range of pixel values with an increasing gamma value.

However, it should be noted that when the original pixel value was high, the pixel value did not decrease significantly even if the *γ* increased because of the non-linear mapping of gamma correction. In other words, if the *γ* is greater than 1, gamma correction will map most of the pixel values close to 0, and only a few with high pixel values will remain close to the original. This means that gamma correction can be used to accentuate target objects with high pixel intensity and significantly reduce unnecessary noise. This notion can be seen in Fig 3(c) and 3(e). Fig 3(c) shows the result of converting the given image (see Fig 3(a)) into the L*a*b* color space and applying the gamma correction with *γ* = 10 to the original a* channel. Unlike before, the target rear-lights region (high intensity) is more prominent with reduced surrounding noise (low intensity). Fig 3(e) shows the result of applying the Otsu threshold method to this refined a* channel. Unlike when thresholding was performed without applying gamma correction, the threshold algorithm appropriately distinguished the object to be extracted and other backgrounds much more easily. Fig 4(b) shows the histogram after applying gamma correction to the a* channel. Compared to Fig 4(a), most of the pixel values are distributed close to 0, and the other pixels are quite far from the dense point. Since the two groups are more clearly separated in the distribution, the Otsu threshold method can easily find a threshold value that separates the target object and the noise region (only some important and detailed elements were classified as objects).

In summary, we perform noise reduction by applying gamma correction to the a* channel, and then conduct thresholding on the refined channel map to extract rear-lights candidate regions.

#### Symmetry test

**Fig 6:**
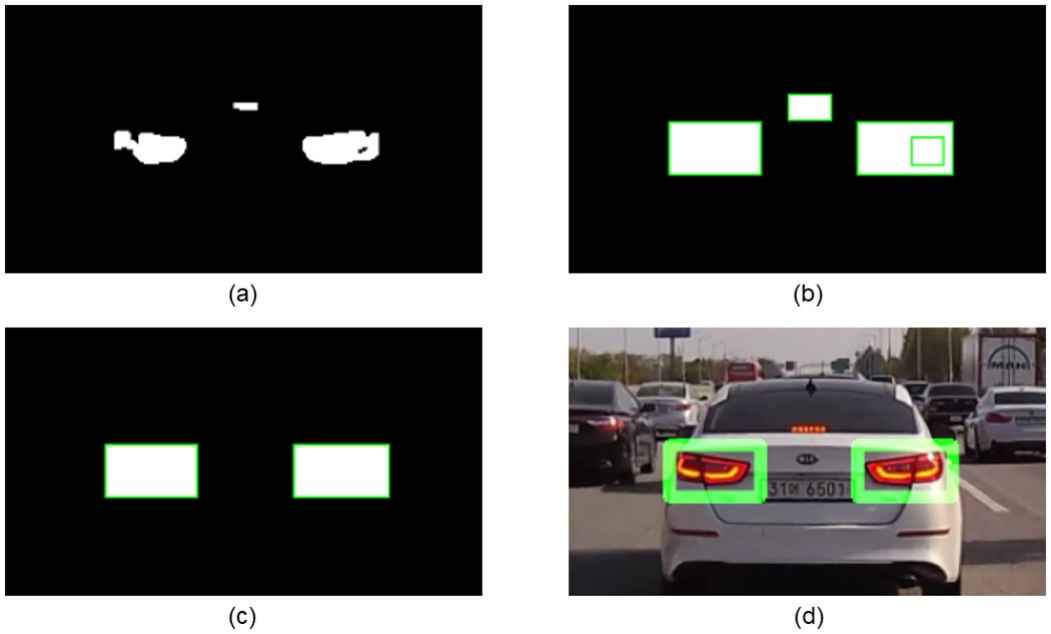
Symmetry test result. (a) Result of applying the masking operation and the morphological operation to the binary image to which thresholding is applied. (b) Result of drawing bounding boxes based on the remaining pixels. (c) Result of finding the optimal rear-lights pair among the candidates through the symmetry test. (d) The final result of rear-lights region detection.

After deriving rear-lights candidate regions, unnecessary regions were masked out using a triangle mask generated based on lane detection. Additionally, the morphological operation was performed to make the remaining pixels appear more clearly. The results of this process are presented in Fig 6(a) and 6(b). However, several unnecessary regions remained after simple filtering. Therefore, it was necessary to find the optimal region of rear-lights in the remaining areas through the verification process of rear-lights. Hence, the property of rear-lights—both pairs of rear-lights of a vehicle are symmetrical—was used. Some studies found the appropriate pair of rear-lights by comparing the pixel-level similarity of each region [15, 19, 23]. Inspired by these methods, we find the optimal rear-lights pair by comparing the correlation coefficient between each candidate region. The correlation coefficient of the two candidate regions can be obtained as follows:

$$\begin{array}{c} sim\left(C_{i},C_{j}\right)=\frac{S_{C_{i}C_{j}}}{S_{C_{i}}S_{C_{j}}} \end{array}$$ (2)

$$\begin{array}{c} S_{C_{i}C_{j}}=\sum_{x}\left(C_{i}\left(x\right)-\mu_{C_{i}}\right)\left(C_{j}\left(x\right)-\mu_{C_{j}}\right) \end{array}$$ (3)

$$\begin{array}{c} S_{C_{i}}S_{C_{j}}=\sqrt{\sum_{x}{\left(C_{i}\left(x\right)-\mu_{C_{i}}\right)}^{2}\sum_{x}{\left(C_{j}\left(x\right)-\mu_{C_{j}}\right)}^{2}} \end{array}$$ (4)

**Fig 7:**
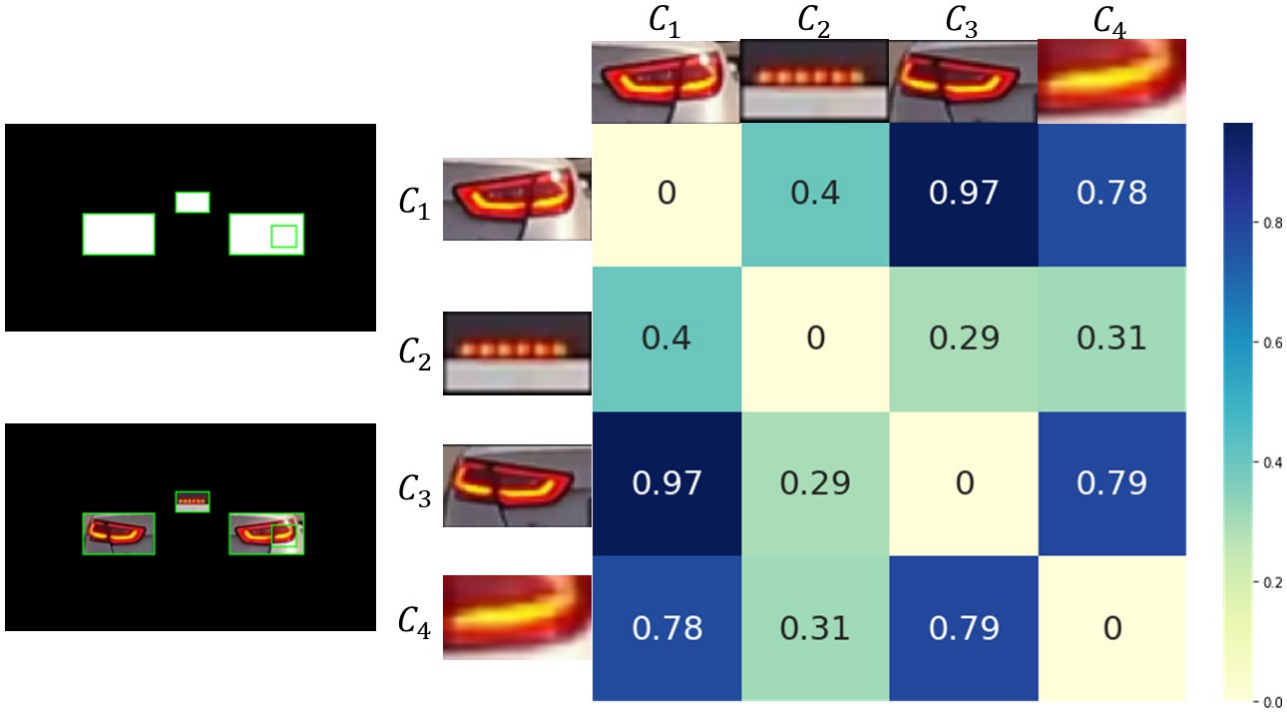
Calculation of pixel-level similarity between two candidate regions using correlation coefficients.

In Eq (2), *sim*(*C*_*i*_, *C*_*j*_) denotes the degree of similarity between *C*_*i*_ and *C*_*j*_ based on the correlation coefficient. *C*_*i*_ and *C*_*j*_ denote candidate regions in the original image. In Eqs (3) and (4), $\mu_{C_{i}}$ and $\mu_{C_{j}}$ represent the mean pixel values of each region. An example of calculating the similarity for each pair of candidate regions is shown in Fig 7. In Fig 7, *C*_1_ and *C*_3_ showed the highest similarity at 0.97, and the selected *C*_1_ and *C*_3_ were the rear-lights pairs we wanted to extract. A simple filtering based on the aspect ratio was conducted to prevent mismatching, which is to not consider a pair if the height and width differ significantly between candidate regions [9, 13, 15, 17]. After applying the symmetry test, the final selection of rear-lights pairs and detection results are both presented in Fig 6(c) and 6(d).

In summary, our rear-lights region detection algorithm consists of color space conversion, noise reduction using gamma correction, thresholding to extract rear-lights candidate regions, and symmetry test to find optimal rear-lights pairs. Specifically, our proposed noise reduction technique makes our algorithm robust to noise, enabling it to effectively derive the rear-lights region of preceding vehicles in various environments.

### Brake-lights detection

**Fig 8:**
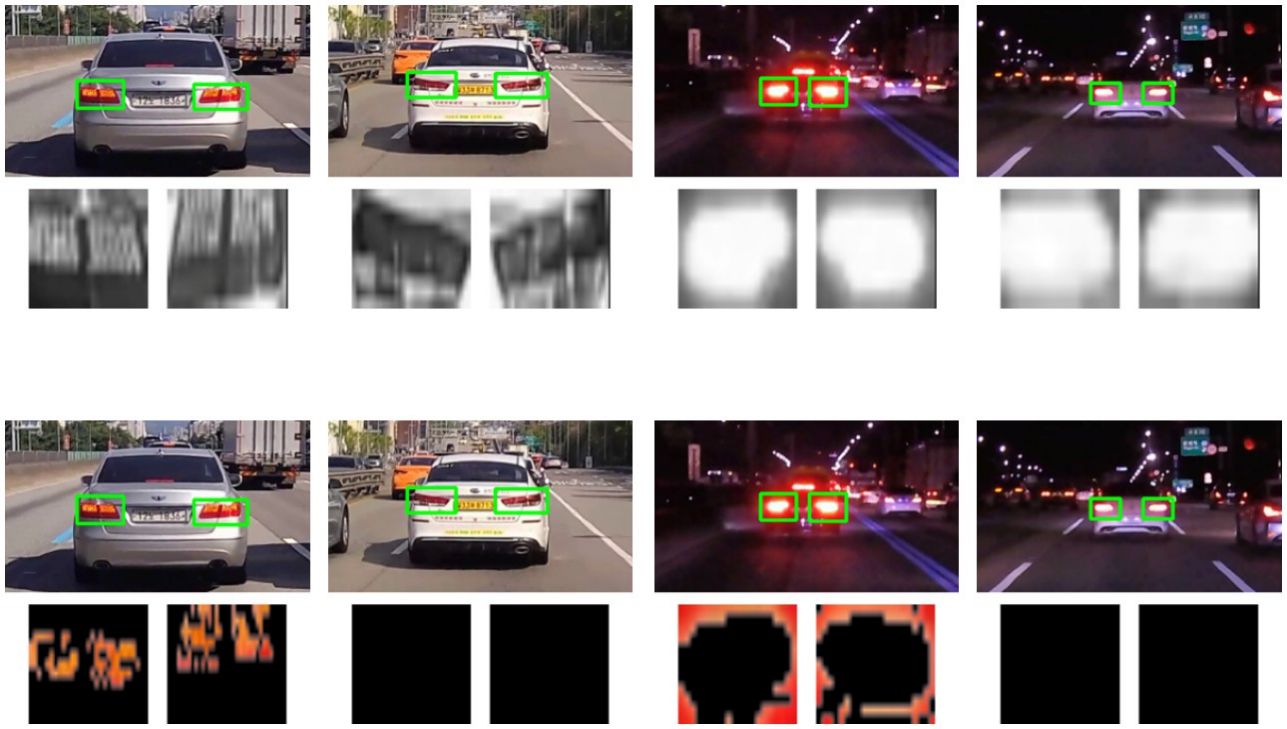
Rear-Lights region detection results and feature extraction of detected rear-lights regions. From the top, the first row is a grayscale image of rear-lights regions, and the second row is the rear-lights regions image with HSV color range filtering applied. From the left, the first column is brake on condition at daytime, the second column is brake off condition at daytime, the third column is brake on condition at nighttime, and the fourth column is brake off condition at nighttime.

We successfully extracted the region of rear-lights of the front vehicle via the method mentioned before. We now need to find the characteristic that can distinguish the on and off conditions of brake based on the derived rear-lights region. Generally, the brightness is greater than when the brake is on than when it is off. Therefore, one can think of it as being able to distinguish between an on and off condition based on the intensity of brightness [15, 19]. Therefore, the brake on and off conditions were classified by converting the derived rear-lights regions into grayscale images and comparing the average intensities. The results are shown in Fig 8. In the first row of Fig 8, there was no significant difference between the grayscale images of the brake on and off conditions at daytime. This is because various noise lights, including sunlight, reflected from the vehicle in the daytime cause the brake off condition to have the same brightness intensity as the brake on condition in the grayscale image even when the brake is off. Furthermore, the first row of Fig 8 shows that it is impossible to distinguish the brake on condition from the brake off condition in nighttime using the brightness intensity because the rear-lights regions of the vehicle are lit even in the brake off condition. Therefore, brightness intensity cannot be an appropriate indicator to distinguish between brake on and off conditions in various environments.

**Fig 9:**
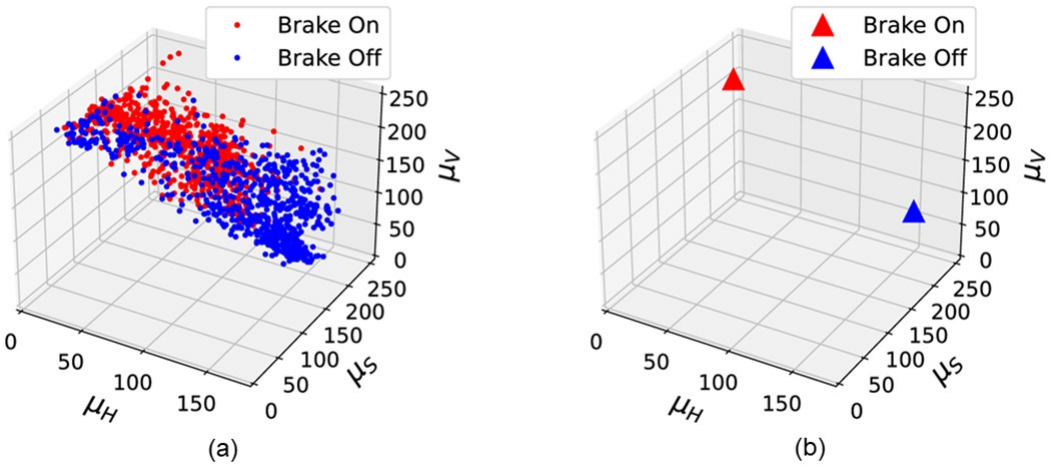
Comparing the mean of HSV channels under brake on and off conditions. (a) Mean of each HSV color channel under brake on and off conditions. (b) Maximum density point for each distribution of brake on and off conditions.

This study focuses on color features to classify brake on and off conditions. Although the method using brightness intensity could not properly distinguish the brake on and off conditions, it was clear that the two conditions had different characteristics. We checked if there were unique color features that could be only attributed to the brake on condition, unlike the brake off condition, in various color ranges. The HSV color space was used to adjust the color range. HSV color space can better represent the way humans observe colors. It consists of a cone-shaped space created by three components: H (Hue), indicating the type of color; S (Saturation), indicating the vividness of color; V (Value), indicating the brightness of color [21]. We compared the mean of each HSV channel in the brake on and off conditions with random samples to extract the color features that appear mainly in the brake on condition. The result is shown in Fig 9. *X*-axis, *Y*-axis, and *Z*-axis represent the mean of the H channel, S channel, and V channel, respectively (Fig 9). The mean of each HSV channel in the brake on and off conditions appeared to differ. The mean V channel value in the brake on condition was highly concentrated between 200 and 250, while the mean V channel value in the brake off condition was sparsely concentrated. Furthermore, the mean value of the H channel in the brake on condition was concentrated between 0 and 50; however, the mean H channel value in the brake off condition was concentrated at a larger point close to 150. The mean S channel values in the two conditions did not show significant difference; both were concentrated above 100. As shown in Fig 9(b), these are more evident when plotting the maximum density point of the distribution in the brake on and off conditions. Based on the comparisons, we set the hue in the range of 0-30, the saturation in the range of 130-255, and the value in the range of 220-250 to extract the color features in the brake on condition.

**Fig 10:**
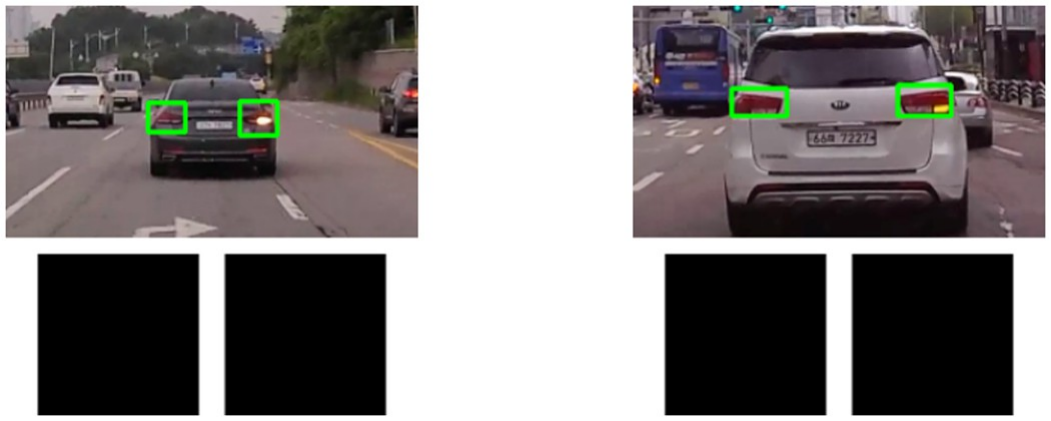
Rear-lights regions image with HSV color range filtering applied when turn signals are on.

The result of applying HSV color range filtering is shown in the second row of Fig 8. The difference between brake on and off conditions became apparent due to color features extracted using HSV color space, unlike when brightness intensity was used. Color features within a specific HSV color range between bright orange and red were clearly visible in the brake on condition but not in the brake off condition. These results also solve the noise problems, such as sunlight in daytime, which were problems when using brightness intensity, and the problem that it was difficult to distinguish between brake on and off conditions at nighttime. In addition, the method using the HSV color range does not extract any features even when the turn signal of the vehicle in front is on, as shown in Fig 10.

**Fig 11:**
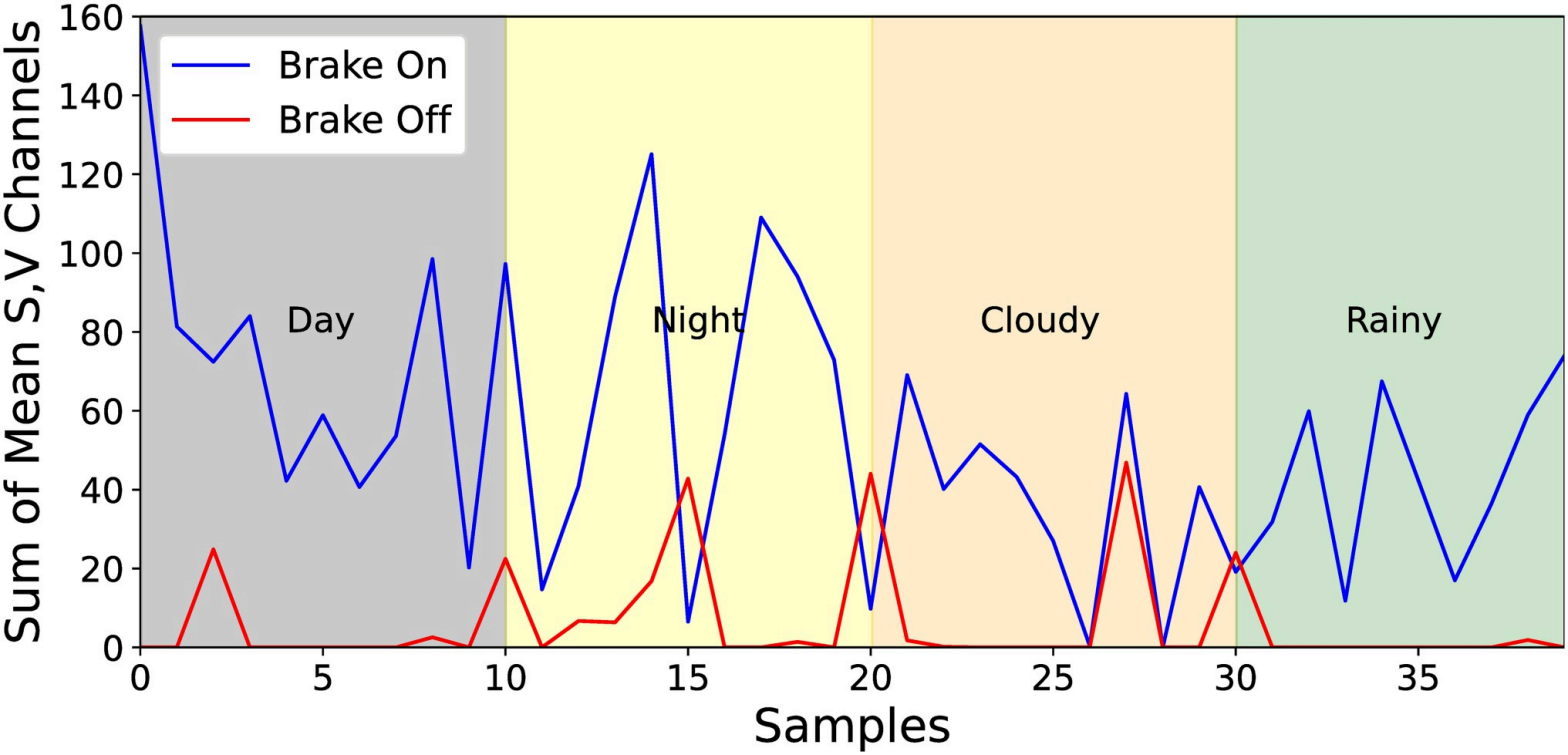
Sum of mean S channel value and V channel value in the brake on and off conditions.

The difference between the brake on and off conditions was verified by averaging the remaining S or V channels without additional conversion or processing for the rear-lights region filtered by the color range. Most of the mean values were close to zero or had a small value in the brake off condition because of filtering performed, whereas the mean value had a larger value in the brake on condition. Additionally, the distribution of the mean value of each condition was relatively more evenly spread than when the brightness intensity was used. The sum of the mean of S and V channels was used as the final decision value as shown in Fig 11. The decision value and the final classification process are as follows:

$$\begin{array}{c} d=\mu_{S}+\mu_{V} \end{array}$$ (5)

$$\begin{array}{c} B=\{\begin{array}{cc} 1, & \text{if}\;d\geq \tau ,\\ 0, & \text{otherwise} \end{array} \end{array}$$ (6)

In Eq (5), *d* denotes a decision value used to determine brake on and off conditions. Furthermore, *μ*_*S*_ and *μ*_*V*_ represent the mean of S and V channels, respectively. In Eq (6), *B* show if the brake is on or off, and *τ* denotes the threshold value. If the decision value *d* was greater than or equal to *τ*, it was considered as a brake on, and if it was less than *τ*, it was classified as brake off.

In summary, our brake-lights detection algorithm converts the derived rear-lights region to HSV color space, applies color range filtering, and then computes the decision value from the remaining pixels. The calculated decision value is compared with the threshold value to finally classify brake on or off conditions. The color range and pre-defined threshold value were set considering applicability in various environments.

## Experimental results

In this section, we evaluate the performance of the proposed method. We evaluated the proposed method for rear-lights region detection and brake-lights detection, respectively. We used an INAVI V300 as our single monocular dashboard camera, which has 1280×720 resolution and 30 FPS. The dashboard camera was mounted at the top center of the test vehicle’s front windshield. The test vehicle is the Volkswagen Tiguan Allspace, with a length of 4.73 m, a width of 1.84 m, and a height of 1.67 m. We collected videos from urban roads and city highways in Seoul, Korea from April 13 to July 23, 2021. We collected 10,991 videos via a forward-facing dashboard camera mounted on a vehicle for the evaluation. Each video was approximately 4 to 5 minutes long and consisted of about 1,800 frames. The collected videos were varied in terms of the vehicle type of the preceding vehicle, the distance between the vehicles, and the lighting condition. Therefore, the collected data was appropriate to evaluate the method in the real driving environment. We classified the collected videos into four environmental categories—day, night, cloudy, and rainy to evaluate them in various environments. To evaluate rear-lights region detection performance, we selected three videos from each category as evaluation videos. It would be meaningless to evaluate the performance of the rear-lights region detection algorithm if the car in front does not exist in the evaluation video, such as on a quiet road or when the cars in front constantly change lanes. Therefore, we manually selected videos that a car must exist in front of the entire video frame for reliable evaluation. To evaluate brake-lights detection performance, we extracted a total of 12,000 frames from each category of collected videos. We extracted 4,000, 4,000, 2,000 and 2,000 frames for the day, night, cloudy and rainy environments, respectively. Similarly, we selected frames with a car present in front of the same lane and within an appropriate distance range for reliable evaluation of brake-lights detection performance. In all the environmental categories, the ratio of brake on and off conditions was equal. We divided the extracted 12,000 frames into 8,600 training data (70%) and 3,400 test data (30%), respectively. Training data is used when model training is required or searching optimal threshold values, and all algorithms including ours were evaluated through the test data.

We conducted comparative evaluation with three existing methods to further explore the effectiveness of our method. The first and second methods are similar to ours in that they extract rear-lights candidate regions through thresholding in the color channel of the input image. However, the first method used **F**RST to find **R**adial symmetric blobs and performed brake-lights detection based on the number of remaining pixels [13] (indicated as **FR**). In the second method, rear-lights region detection was performed through the **H**orizontal-vertical peak value verification process, and brake-ligths detection was performed by training a **S**VM [49] based on six features extracted from the derived rear-lights region [10] (indicated as **HS**). The third method performed rear-ligths and brake-ligths detection simultaneously based on deep learning object detection networks called **YO**LOv3-tiny [43], and used an additional output layer, spatial pyramid pooling (SPP) layer, and focal loss to improve performance [37] (indicated as **YO**).

### Results of rear-lights region detection

First, we evaluated the performance of the rear-lights region detection algorithm. We used the detection rate (DR) as an evaluation metric according to standard in literatures [9, 13, 14, 23]. DR is calculated for selected evaluation videos. DR measures how well the algorithm detects the rear-lights region pair present in each frame of a given video. Specifically, DR refers to the percentage of rear-lights regions detected by the algorithm out of all ground-truth rear-lights regions present in a given video. We considered each pair of left and right rear-lights region as one ground-truth. Hence, there were a total of two ground-truths in one frame. We used the DR concept to evaluate our rear-lights region detection algorithm on a given video for each environmental category.

The evaluation results are presented in Table 1. Our rear-lights region detection algorithm showed quite high performance in all scenarios. Particularly, the total DR was as high as 93% and 92% in a day and cloudy scenarios, respectively. Despite the presence of severe surrounding noise in the day scenario and low visibility in the cloudy scenario, the experimental results show that our algorithm is robust to such noise and environmental changes. Additionally, our algorithm showed a total DR of 89% even in a night scenario with strong light noise from surrounding vehicles and low visibility, indicating that our algorithm is easily applicable at nighttime and has strong noise robustness. Finally, our algorithm showed a total DR of 87% in a rainy scenario. Rainy scenario has worse visibility because of raindrops and vehicle’s wiper operation. However, our algorithm is still robust against such difficulties.

**Table 1:** Rear-lights region detection algorithm evaluation result.

Scenario	Video Index	Ground Truth	Correct Detection	DR (%)	DR (Total) (%)
Day	1	3600	3517	97.69	93.96
	2	3598	3336	92.71	
	3	2302	2073	90.05	
Night	1	3958	3609	91.18	89.36
	2	3600	3219	89.42	
	3	3600	3143	87.31	
Cloudy	1	3602	3539	98.25	92.45
	2	3600	3263	90.64	
	3	3602	3186	88.45	
Rainy	1	3600	3361	93.36	87.56
	2	2444	2056	84.12	
	3	3600	3026	84.06	

**Fig 12:**
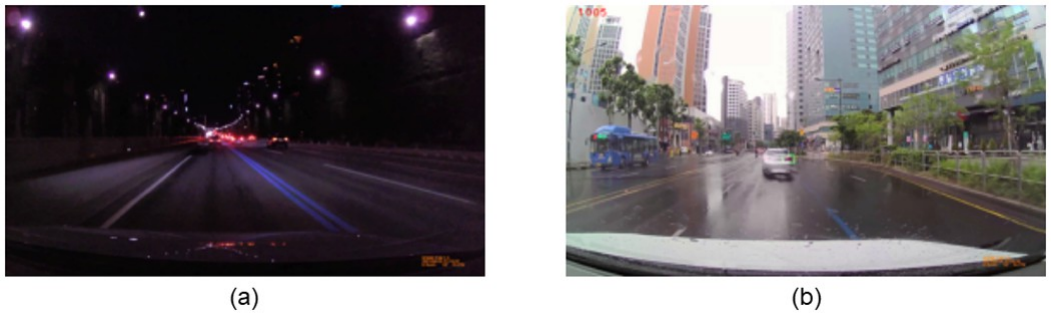
Rear-lights region detection failure cases. (a) Failed to detect rear-lights region due to the distance from the vehicle in front. (b) Failed to detect rear-lights region due to raindrops.

The main reasons for the failure of rear-lights region detection are presented in Fig 12. Fig 12(a) shows a situation where the rear-lights region detection fails because the distance from the vehicle in front was very far. When the distance from the vehicle in front was very far, the algorithm found it very difficult to extract red color features present on the given frame. Even if they were extracted, they would have been considered as simple noise because they were quite small. However, the algorithm extracted the rear-lights region well at an appropriate distance, unless the distance from the vehicle in front was very far. Fig 12(b) shows a situation where rear-lights region detection failed due to raindrops on the windshield of a vehicle on a rainy scenario. In this case, the rear-lights regions of the front vehicle were blurred, causing the algorithm to fail the detection, or it extracted only one region of the rear-lights. This situation became more pronounced on days with heavy rains. However, the algorithm successfully extracted rear-lights regions again after a few frames have passed when either raindrops were flowing or the wiper wiped them off.

The comparative evaluation results for the algorithm are presented in Table 2. Compared to our method, the FR and HS methods showed significantly lower performance. This is because both FR and HS methods conducted vehicle detection before rear-lights region detection. In both methods, a compact image with a clear rear view of the vehicle was extracted from the raw image as a result of vehicle detection, and rear-lights region and brake-lights detection was performed under the refined image. This additional process effectively removes surrounding noises, but it requires model training for vehicle detection and increases computational cost. Therefore, although the two methods performed well for the refined images, they did not work well in the noisy raw environment like the data we collected. In addition, we evaluated the FR and HS methods by adding the gamma correction process for noise reduction used in our algorithm (indicated as **FR+GC**, and **HS+GC**). As shown in Table 2, the FR and HS methods with gamma correction significantly improved the performance. This means that the gamma correction process can reduce noise and increase the performance of rear-lights region detection. In other words, the gamma correction process has the effect of refining the raw image without additional vehicle detection process. Finally, the YO method showed high performance similar to ours, especially in the night scenario. However, our algorithm is more efficient considering that YO uses deep learning models which require a huge amount of training data/time and have high computational cost.

**Table 2:** Comparative evaluation result of rear-lights region detection algorithm.

Method	Approach	Scenario (DR (%))			
		Day	Night	Cloudy	Rainy
Our	F-based	**93.96**	89.36	**92.45**	**87.55**
FR [13]	F-based	5.26	18.07	13.18	12.83
HS [10]	F-based	3.77	19.94	12.46	15.45
FR [13] + GC	F-based	60.12	79.11	78.83	72.03
HS [10] + GC	F-based	68.02	65.25	71.80	63.70
YO [37]	DL-based	80.88	**93.49**	86.21	86.67

**Fig 13:**
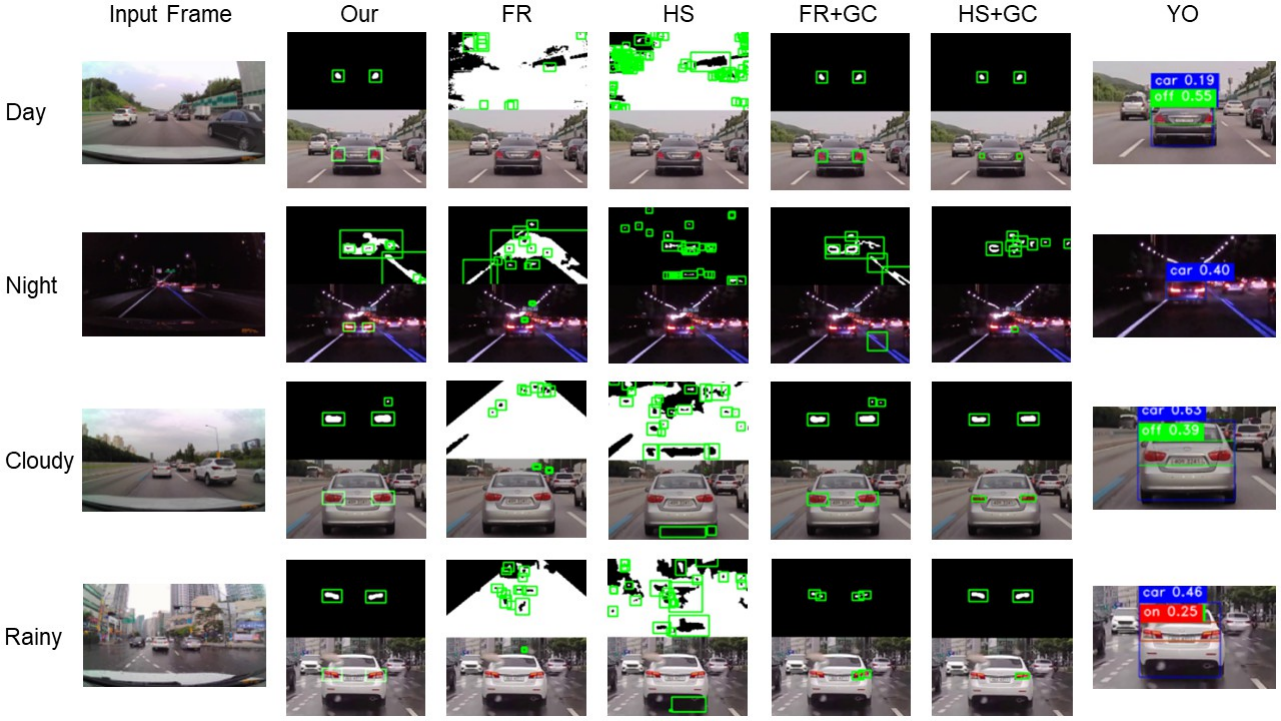
Comparative evaluation result of rear-lights region detection algorithm.

Examples of rear-lights region detection results for all methods are shown in Fig 13. Compared with ours, the thresholded images of the FR and HS methods contained a lot of noise, and as a result, rear-lights region detection was not performed successfully. On the other hand, in the FR and HS methods with gamma correction, it can be seen that the noise in the thresholded images is significantly reduced, and the rear-lights region detection result is also improved. Nevertheless, the FR+GC and HS+GC methods failed to derive the correct rear-lights regions in the night and rainy scenarios, even with similar noise levels to ours. This seems to be due to the difference in the method of finding the optimal rear-lights pair in the remaining candidate regions. Finally, in the case of the YO method, detection was generally successful. However, as seen in the night scenario, detection often failed when the distance from the preceding vehicle increased.

In summary, comparative evaluation results in Table 2 and Fig 13 show that the gamma correction we used was effective in improving the rear-lights region detection performance by reducing the noise in the input image. Additionally, the results prove that our method showed good rear-lights region detection performance in a situation similar to the real driving environment.

### Results of brake-lights detection

We evaluated the performance of the proposed brake-lights detection algorithm. The brake-lights detection algorithm determines whether the preceding vehicle in a given frame is in a brake on or off condition. We evaluated our brake-lights detection algorithm on selected test data. In addition, we compared the performance of our method with the three existing methods used in the evaluation of rear-lights region detection, and only considered the brake-lights detection algorithm.

For evaluation metrics, recall score and precision score were used considering two different functions of CAS. The formulas for recall and precision scores are as follows:

$$\begin{array}{c} recall=\frac{TP}{TP+FN} \end{array}$$ (7)

$$\begin{array}{c} precision=\frac{TP}{TP+FP} \end{array}$$ (8)

In Eqs (7) and (8), *TP* denotes true positive, meaning that the brake of the vehicle in front is actually on, and the algorithm predicts correctly. In Eq (7), *FN* represents false negative, meaning that the brake of the vehicle in front is actually on, but the algorithm incorrectly predicts otherwise. In Eq (8), *FP* represents false positive, meaning that the brake of the vehicle in front is off, but the algorithm erroneously predicts that the brake of the vehicle in front is on. As mentioned earlier, CAS should send a warning to the driver or automatically apply the brakes without the input from the driver in an expected collision situation [2]. If the brake-lights detection algorithm is used for CAS such that it only alerts the driver in an expected situation of a collision, sending an erroneous warning when the brakes of the vehicle in front are not turned on do not pose a big problem. It is because the driver can acknowledge the warning and reconsider the situation by self-checking. Hence, the algorithm should focus on finding as many situations as possible when the brakes of the car in front are on. Recall score is suitable for this case because it evaluates the number of situations the algorithm can predict where the brakes are applied when the vehicle in front actually applies the brakes. However, if the detection algorithm is used in CAS that automatically applies the brakes without providing input to a driver in an expected collision situation, sending a false warning to the driver when the brakes of the vehicle in front are not turned on can lead to an accident as dangerous as a collision [50]. Therefore, it is necessary to accurately predict the brake condition of the vehicle in front while simultaneously considering the degree of false positive carefully. The precision score is suitable in this case as a metric that evaluates the number of situations in which the brake of the car in front is actually applied in the situation where the algorithm predicts that the brake of the car in front is applied. Therefore, we considered both the recall score and the precision score to evaluate the performance of the brake-lights detection algorithm.

**Fig 14:**
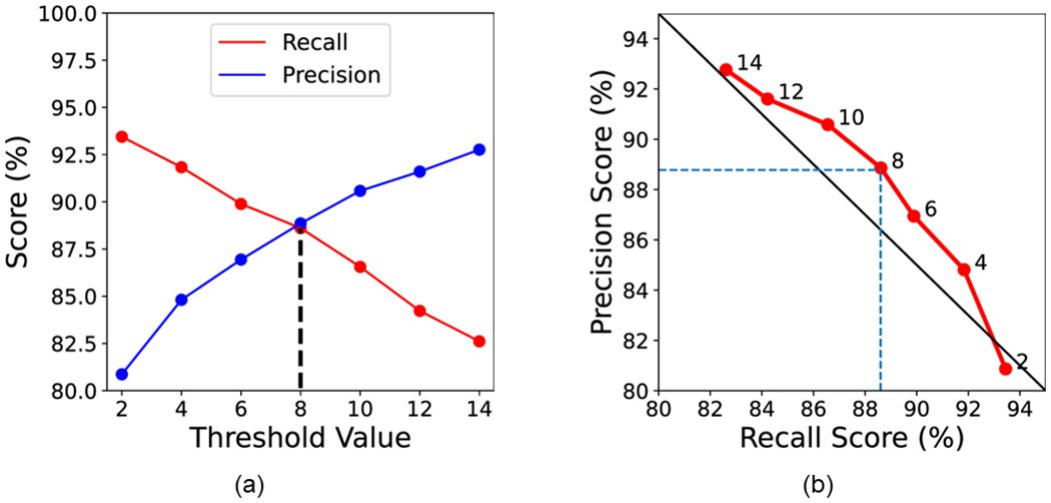
Relationship between recall score and precision score. (a) Recall score and precision score were evaluated while changing the threshold value. (b) Precision-recall curve (PR curve).

We set the optimal threshold value *τ* to 8 as a result of experimenting with several training samples from each category. Moreover, we also obtained evaluation results by adjusting the threshold value considering the trade-off relationship between recall score and precision score. The trade-off relationship between recall score and precision score when evaluating while changing the threshold value is shown as a graph in Fig 14(a). It provide possibility to improve the flexibility of the system by adjusting the threshold value based on the purpose of the CAS, sending a warning to the driver, or automatically applying the brake. Additionally, Fig 14(b) presents the precision-recall curve showing the trade-off relationship between the two scores. The black line denotes the reference line, and the red curve shows the recall score and the corresponding precision score. Each threshold value is annotated on the curve. The precision score decreased slowly with the increasing recall score (see the red curve in Fig 14(b)). Moreover, as the threshold value passed through 8 and 6, the decreasing width increased. That is, the point where the threshold value is 8 was the most concave part of the curve, meaning that, when the threshold value was 8, the overall performance of the precision score and recall score was the best. Therefore, the optimally selected threshold value of 8 is an appropriate value considering both the precision score and recall score.

**Fig 15:**
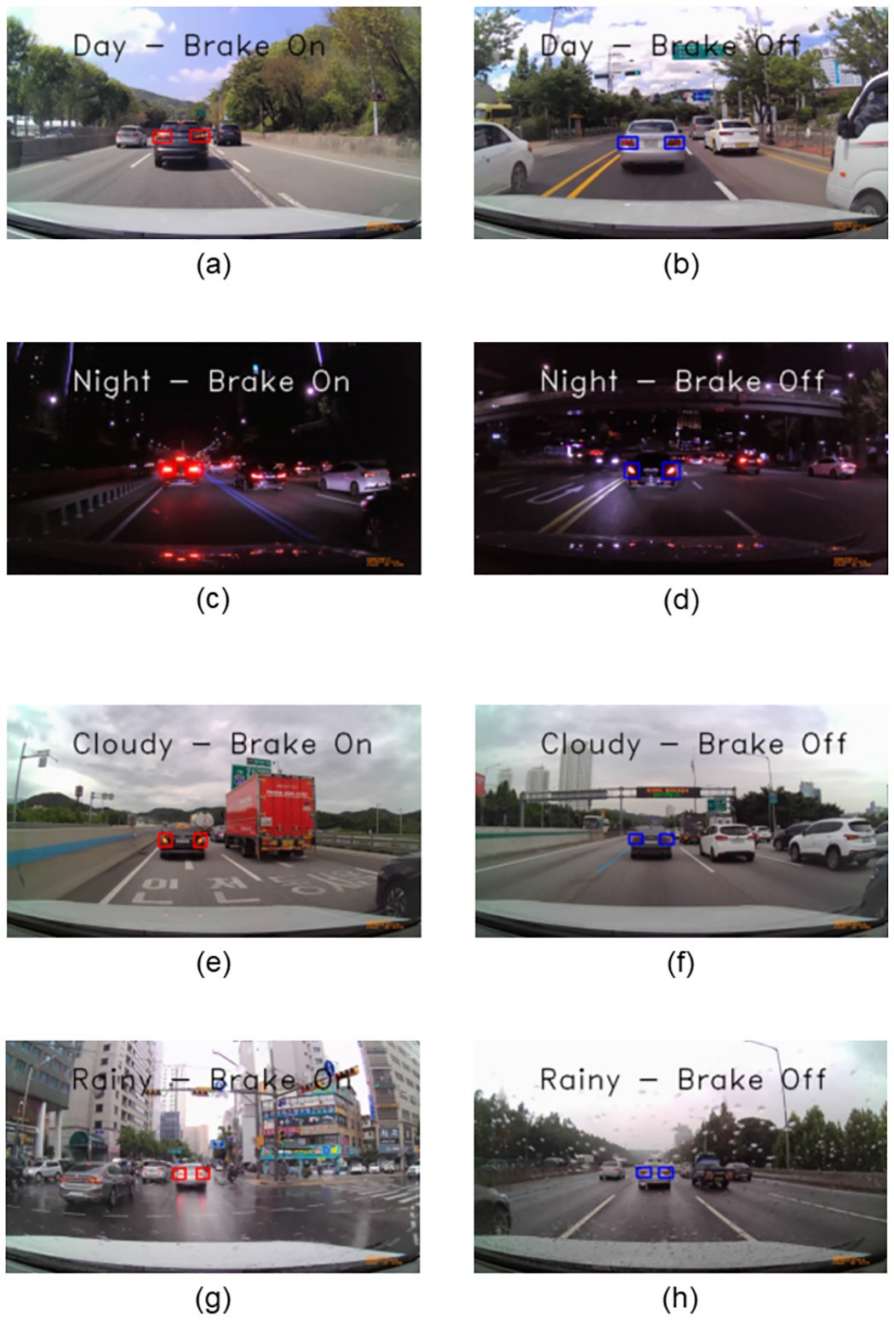
Brake-lights detection result. (a) Brake on condition in a day environment. (b) Brake off condition in a day environment. (c) Brake on condition in a night environment. (d) Brake off condition in a night environment. (e) Brake on condition in a cloudy environment. (f) Brake off condition in a cloudy environment. (g) Brake on situation in a rainy environment. (h) Brake off condition in a rainy environment.

Examples of performing brake-lights detection are shown in Fig 15. The images in the left of Fig 15 are cases of successful prediction of the brake on condition of the vehicle in front, and the rear-lights region is marked with a red rectangle. Conversely, the images in the right of Fig 15 are cases of successful prediction of the brake off condition of the vehicle in front, and the rear-lights region is marked with a blue rectangle.

Table 3 shows the performance of our proposed brake-lights detection algorithm and the performance comparison results with other methods. In Table 3, our algorithm showed good performance with a recall score of about 88.6% and a precision score of about 88.9% in the test data, and the precision score and recall score were in balance. The second row of Table 3 is the result of classifying the brake on and off conditions based on the mean brightness intensity in the grayscale image, which is also mentioned in previous section (indicated as **Our+BI**). As a result, both the total recall score and precision score were very poor. The imbalance and irregularity of the two scores were more severe than ours. This indicates that it is difficult to distinguish between brake on and off conditions with mean brightness intensity due to noise caused by ambient light and weather conditions. It is even more difficult to apply it to various environments. In the case of FR method, the performance was slightly better than Our+BI, but the imbalance between recall and precision was deepened. This means that the method using FRST and radial symmetry blobs used in the FR method is difficult to be globally applied to various environments. This low environmental applicability deteriorates the total performance of FR. The HS and YO methods perform brake-lights detection in a learning-based method using SVM and YOLOv3-tiny, respectively. As a result, both methods showed high performance, especially the YO method was slightly higher than ours. However, it should be noted that both methods require model training. Our method does not use any trained model, but our method outperforms the HS method and is highly comparable to the YO method. Considering the performance gap between Our+BI/FR and HS/YO, it can be seen that our method improves performance by successfully mitigating the noise problem and applicability problem faced by previous feature-based methods.

**Table 3:** Brake-lights detection algorithm evaluation result.

Method	Approach	Training	Metrics	Scenario				
				Day	Night	Cloudy	Rainy	Total
Our	F-based		Recall (%)	93.33	82.83	85.00	94.33	**88.61**
			Precision (%)	89.46	85.25	91.40	92.18	**88.86**
Our+BI	F-based		Recall (%)	44.17	99.67	47.33	66.67	66.28
			Precision (%)	81.79	57.95	81.14	73.73	66.80
FR [13]	F-based		Recall (%)	91.83	84.00	85.67	90.67	87.78
			Precision (%)	56.86	49.17	55.39	53.86	53.55
HS [10]	ML-based	✓	Recall (%)	80.83	97.17	76.00	88.00	86.67
			Precision (%)	85.09	84.86	88.03	87.71	85.86
YO [37]	DL-based	✓	Recall (%)	82.17	97.50	87.67	88.33	**89.22**
			Precision (%)	86.64	92.27	86.23	89.23	**88.98**

Table 4 shows the comparison results of processing speed of our algorithm and the YO method using a deep learning-based approach. The YOLOv3-tiny model used in the YO method is famous for its lightweight and fast network, but it could only process about 6 frames per second. However, our algorithm was able to process about 37 frames per second and showed similar performance to the YO method. It means that our algorithm is sufficient for real-time CAS and it is efficient in terms of computational cost. Table 5 shows the ratio of the average time required for each process to the total time required. In Table 5, LD, RD, and BD refer to an ROI setting algorithm using lane detection, rear-lights region detection, and brake-lights detection, respectively. The ROI setting process using the lane detection occupied approximately all of the total process duration. Therefore, if a more efficient and powerful lane detection algorithm than used in this paper is used, the overall computational cost will be much lower. The evaluation process was performed on the OpenCV 4.5.2 version with a Python interface. The evaluation environment consisted of MS Windows 10 64-bit OS, AMD Ryzen 7 5800X 8-Core CPU @3.80 GHz, and 32GB RAM.

**Table 4:** Comparison of processing speed.

Method	Approach	Training	FPS
Our	F-based		**37**
YO [37]	DL-based	✓	6

**Table 5:** Proportion of time required by algorithms.

Algorithm	Proportion (%)
LD	**99.8**
RD	0.1
BD	0.1

## Conclusion

This paper proposes a vision-based approach to implement CAS specialized for rear-end collision situations. First, we set an ROI that applied the lane detection to consider only the information of the vehicle in front in the same lane for a system more suitable for rear-end collision situations. Next, we presented a rear-lights region detection algorithm that can extract the rear-lights region of the vehicle in various environments to solve the low applicability and noise sensitivity problems in various environments encountered by the existing feature-based approach. Finally, we presented a break-lights detection algorithm based on color features extracted from HSV color space to determine whether the front car brakes in various environments. Experimental results indicate that both the rear-lights region detection algorithm and the brake-lights detection algorithm show high performance in various environments. However, our proposed methods conducted rear-lights region detection and brake-lights detection in a frame-by-frame manner. The preceding vehicle’s rear-lights region or braking state may appear consistent in adjacent frame sets. Therefore, our future study is to use a tracking algorithm (i.e., Kalman filter) to further boost the performance of our method under these strong consistency assumptions. In addition, additional considerations may be required to improve performance in a nighttime environment where performance is relatively low compared to other environments. As a solution, we can either use shape or edge features that are less sensitive to light noise or design a more strict verification algorithm for filtering noise blobs. Finally, we will apply a more efficient and faster lane detection algorithm to improve the processing speed of our method.
